# Supplementary material for: Determinants of primary healthcare providers’ readiness for integration of ART services at departmental levels: A case study of Lira City and District, Uganda
Source: PLoS One. 2023 Oct 5;18(10):e0292545. doi: 10.1371/journal.pone.0292545 (PMC10553216; doi:10.1371/journal.pone.0292545)
Supplement: Attachment — Submitted filename: Reviewer comments_v1.docx [file pone.0292545.s002.docx]

**Determinants of primary healthcare providers’ readiness for integration of ART services in departmental levels: A case study of Lira City and District, Uganda**

I was very interested to review this manuscript and found relevant as far as the health system is concerned in today’s resource context. Here, I’ve highlighted my comments to be seen by authors for the improvement of this manuscript before publication.

Abstract

- Results: It would be better if you incorporate the 95% confidence interval for the level of readiness for integration of ART services at departmental levels.
- Conclusions and recommendations:
  - Avoid the term ‘recommendations’ since the conclusions are meant to also consider the potential recommendations.
  - Conclusions for relevant determinants not included

Introduction

- The narration “The current success in the effectiveness of the vertical programs has been attributed to the donor funds [4]. In Uganda and across the region, HIV services are provided by vertically operating separately from other health system functions [5]. Notwithstanding the achievements and triumphs already realized, several questions around its sustainability are becoming very critical.” (Page 3 line numbers 73-77) is a duplication of description (page 3 line numbers 57-61). Please reconsider it.
- The authors didn’t mention the potential determinants of providers’ readiness to service integration approach identified by previous studies. It seems only a baseline study of the magnitude or level of readiness to such integration issue. The authors should incorporate such point somewhere here in the introduction section. Otherwise, avoid the determinants in the whole manuscript including the title.
- What is known about the level of integration as well as the determinants should be highlighted clearly.

Materials and methods

- Primary healthcare providers versus the health facilities. Which one was the focus of this study? (Page 4 line numbers 98-102)
- The qualitative part lacked important details for example data analysis software, how trustworthiness managed, who collected data and how many themes developed.

Discussion: It would be better to add more similar empirical evidence in the literature to make the discussion more appropriate.
