# Supplementary material for: Determinants of primary healthcare providers’ readiness for integration of ART services at departmental levels: A case study of Lira City and District, Uganda
Source: PLoS One. 2023 Oct 5;18(10):e0292545. doi: 10.1371/journal.pone.0292545 (PMC10553216; doi:10.1371/journal.pone.0292545)
Supplement: Attachment — Submitted filename: Responses to reviewers.docx [file pone.0292545.s003.docx]

Dear Editor,

We wish to resubmit our revised journal article titled the Determinants of primary healthcare providers’ readiness for integration of ART services in departmental levels: A case study of Lira City and District, Uganda. All the supporting files requested has been attached.

We thank you in advance.

Sean Steven Puleh

| **REVIEWERS COMMENTS** | **RESPONSES** | **LINE NUMBER** |
| --- | --- | --- |
| **Abstract** | | |
| - Results: It would be better if you incorporate the 95% confidence interval for the level of readiness for integration of ART services at departmental levels. | Thank you highlighting this gap. We have addressed it by inserting the 95% confidence interval as advised. Readiness was 75.2% (95% CI; 0.703 - 0.795) | Line 29 |
| - Conclusions and recommendations:   - Avoid the term ‘recommendations’ since the conclusions are meant to also consider the potential recommendations. | We are grateful you pointed out this and it has been deleted as advised | Line 38 |
| - Conclusions for relevant determinants not included | We have adjusted the conclusion by adding the necessary determinants. The PHCPs who were knowledgeable and those who had worked for at least 6 months at the current facility were strong determinants of the integration of ART services in resource-limited settings. | Lines 39 - 41 |
| **Introduction** | | |
| The narration “The current success in the effectiveness of the vertical programs has been attributed to the donor funds [4]. In Uganda and across the region, HIV services are provided by vertically operating separately from other health system functions [5]. Notwithstanding the achievements and triumphs already realized, several questions around its sustainability are becoming very critical.” (Page 3 line numbers 73-77) is a duplication of description (page 3 line numbers 57-61). Please reconsider it. | Thank you for detecting the duplication in the write up. We have made the necessary adjustment in the write up | Line 73 - 77 |
| The authors didn’t mention the potential determinants of providers’ readiness to service integration approach identified by previous studies. It seems only a baseline study of the magnitude or level of readiness to such integration issue. The authors should incorporate such point somewhere here in the introduction section. Otherwise, avoid the determinants in the whole manuscript including the title. | Thank you for raising an important part of the introduction. Some of the potential determinants include; the current ART service delivery models, health worker capacity, infrastructure, and financing mechanisms. | Lines 75 - 79 |
| **Materials and methods** | | |
| - Primary healthcare providers versus the health facilities. Which one was the focus of this study? (Page 4 line numbers 98-102) | Thank you for highlighting this error, the focus of the study is on the primary healthcare providers. | Line 103 |
| - The qualitative part lacked important details for example data analysis software, how trustworthiness managed, who collected data and how many themes developed. | Thank you for raising this important aspect of the methodology. We have tried to the best of our ability to address as requested. The data was collected by three research assistants of social science background.  The software used was Nvivo and how the trustworthiness managed has been described. | Lines 140 - 167 |
| **Discussion:** | | |
| It would be better to add more similar empirical evidence in the literature to make the discussion more appropriate. | Much as we appreciate this concern, we were able to get a limited number of empirical data which helped us discuss our findings better. | Lines 434 - 501 |
