# Supplementary material for: Determinants of primary healthcare providers’ readiness for integration of ART services at departmental levels: A case study of Lira City and District, Uganda
Source: PLoS One. 2023 Oct 5;18(10):e0292545. doi: 10.1371/journal.pone.0292545 (PMC10553216; doi:10.1371/journal.pone.0292545)
Supplement: Attachment — Submitted filename: Responses to reviewers.docx [file pone.0292545.s004.docx]

Dear Editor,

We wish to resubmit our revised journal article titled the Determinants of primary healthcare providers’ readiness for integration of ART services in departmental levels: A case study of Lira City and District, Uganda. All the supporting files requested has been attached.

We thank you in advance.

Sean Steven Puleh

| **REVIEWERS COMMENTS** | **RESPONSES** | **LINE NUMBER** |
| --- | --- | --- |
| **Abstract** | | |
| 1. Re-write the 'recommendations for relevant determinants' in your abstract and make the information consistent with what you presented in your response letter. Statement in the response letter: PHCPs who were knowledgeable and those who had worked for at least 6 months (perhaps you wanted to write 6 years) at the current facility, were strong determinants for the integration of ART services in resource limited settings. Lines 39-41 Statement in the abstract: The PHCPs who were knowledgeable are key at achieving integration of ART services at departmental level. Most of the respondents who had worked for at least 6 months (perhaps you wanted to write 6 years) at the current facility were strong determinants of the integration of ART services in resource limited settings. Lines 39-41 | Thank you highlighting these inadequacies in our write up. We have improved it as follow.  The study reveals a high level of readiness for the integration of ART services at departmental levels among Primary Healthcare Providers (PHCPs). Notably, PHCPs knowledgeable about integration and those who spent at least six months at the current health facility of work, were strong determinants for the integration of ART services in resource limited settings. In light of these findings, we recommend that policymakers prioritize the implementation of training programs aimed at upskilling healthcare workers. Furthermore, we advocate that a cluster randomized controlled trial be conducted, to evaluate the long-term effects of this integration on overall health outcomes.   - In this study we meant PHCPs who worked in the current health facility for at least 6 months. | Line 39 - 46 |
| Also, be specific with what 'knowledge' you are referring to, and what you mean with 'current health facility'. If the 'PHCPs' awareness or knowledge on the integration of ART services', was a determinant, be  specific in your communication. Likewise, a decade from now, readers may not understand your term 'current health facility', so be explicit with your description of 'current'. | Thank you for this suggestion. We were able to specify knowledge as  “knowledge on the integration of ART services”  In addition, 'current health facility' was specified to read “at the current health facility of work” | Line 41 - 42 |
| 2. The information on recommendations in the revised abstract is passive, erroneously written, and has lost the impact of a recommendation. Lines 42-46 reads " We recommend that policymakers should consider exploring training the health workers to enhance improving the integration of ART services into departmental levels as a novel path. be conducted to determine the long-term impact of the integration on health outcomes using cluster randomized controlled trials". Please read your detailed recommendations in lines 515-523, and summarize precisely the recommendations in your abstract. | We thank you for highlighting this errors. We improved the recommendation as advised.  It now read: In light of these findings, we recommend that policymakers prioritize the implementation of training programs aimed at upskilling healthcare workers. Furthermore, we advocate that a comprehensive study be conducted, employing cluster randomized controlled trials, to evaluate the long-term effects of this integration on overall health outcomes. | Line 43 - 46 |
| 3. Attend to grammatical and typographic errors in the following statements  - Lines 158-159: A seven-step thematic analysis model by Clerke and Braun was used to analyse qualitative data was analyzed using thematic analysis [21]. | A seven-step thematic analysis model by Clerke and Braun was used to analyse qualitative data using thematic analysis [21]. | Line 171 - 172 |
| - Lines 162: over 15 themes | This was improved to read A total of 15 …. | Lines 174 -175 |
| - Lines 457-460: Our findings are consistent with evidence from elsewhere, which found that integrating HIV services are increased patient satisfaction, increased perceived quality of care, and increased patient access to services and patient health outcomes [24, 25]. | Our findings are consistent with evidence from elsewhere, which found that integrating HIV services increased patient satisfaction, perceived quality of care, and patient access to services, and patient health outcomes [24, 25]. | Lines 473 - 476 |
| - Lines 542-543: Ikwara Emmanuel Asher designed conceptualized, designed the study and designed the study instruments, obtained the grant and necessary approvals.  - Lines 546-548: Sean Steven Puleh conceptualized and designed the study, designed the study instruments, led data collection activities, and substantially contributed to paper development. in addition to providing  mentorship during study conduct. | Ikwara Emmanuel Asher conceptualized the study, designed the study instruments, obtained the grant and necessary approvals. Godfrey Mwesiga, Syliviah Namutebi, Rogers Isabirye and Lakeri Nakero assisted in the conceptualization, design, and conduct of the study. Maxson Kenneth Anyolitho performed the qualitative analysis and contributed to the drafting of the paper. In addition to serving as a mentor while the study was being conducted, Sean Steven Puleh conceptualized the study, designed the study instruments, oversaw the activities for collecting data, conducted the quantitative data analysis, and made significant contributions to the creation of the paper. All authors have read and approved the manuscript and agree to take responsibility and be accountable for the contents of the article. | Lines 561 -569 |
